# Real-time surveillance of surgical margins via ICG-based near-infrared fluorescence imaging in patients with OSCC

**DOI:** 10.1186/s12957-020-01874-z

**Published:** 2020-05-15

**Authors:** Jiongru Pan, Han Deng, Shiqi Hu, Chengwan Xia, Yongfeng Chen, Jianquan Wang, Yuxin Wang

**Affiliations:** 1 Department of Oral and Maxillofacial Surgery, Nanjing Stomatological Hospital, Medical School of Nanjing University, Nanjing, China; 2 Department of Stomatology, The First Affiliated Hospital of Bengbu Medical College, Bengbu, China; 3 School of Medical Imaging, Bengbu Medical College, Bengbu, China

**Keywords:** Surgical margin, Indocyanine green, Near-infrared fluorescence imaging, Oral squamous cell cancer, Quantification

## Abstract

**Background:**

Local recurrence is the main cause of death among patients with oral squamous cell carcinoma (OSCC). This study assessed near-infrared fluorescence (NIF) imaging and spectroscopy to monitor surgical margins intraoperatively for OSCC.

**Methods:**

Cytological and animal experiments were first performed to confirm the feasibility of monitoring surgical margins with NIF imaging and spectroscopy. Then, 20 patients with OSCC were included in the clinical trials. At 6–8 h after 0.75 mg/kg indocyanine green (ICG) injection, all patients underwent surgery with NIF imaging. During the surgery, both NIF images and quantified fluorescence intensity were acquired to monitor the surgical margins.

**Results:**

In cytological and animal experiments, the results showed it was feasible to monitor surgical margins with NIF imaging and spectroscopy. Fluorescence was detected in primary tumors in all patients. The fluorescence intensities of the tumor, peritumoral, and normal tissues were 398.863 ± 151.47, 278.52 ± 84.89, and 274.5 ± 100.93 arbitrary units (AUs), respectively (*P* < 0.05). The SBR of tumor to peritumoral tissue and normal tissues was computed to be 1.45 ± 0.36 and 1.56 ± 0.41, respectively. After primary tumor excision, the wounds showed abnormal fluorescence in four patients (4/20), and residual cancer cells were confirmed by pathological examination in two patients (2/20).

**Conclusion:**

These findings confirmed the complementary value of NIF imaging during radical tumor resection of OSCC. Before tumor resection, we could utilize the fluorescence margin produced by ICG NIF imaging to determine the surgical margin. Moreover, after tumor blocks were removed, the status of surgical margin could also be evaluated rapidly by ICG NIF imaging of tumor bed and in vitro specimens.

## Background

The incidence of oral squamous cell carcinoma (OSCC) has risen in the past decade. Surgical resection is the most common and effective treatment [1]. Unfortunately, remaining cancer cells in the area surrounding the resection can lead to local recurrence. Therefore, the removal of all cancer cells during surgery is critical for the success of the operation. The surgical margin is the only accessible approach to detect remaining cancer cells after excision, which is vital to the prognoses of patients with OSCC [2]. Positive surgical margins are associated with significantly reduced survival rates [3]. Currently, identification of the tumor border is typically dependent on intraoperative visual and tactile examination. After tumor ablation, intraoperative frozen sections are the gold standard to confirm negative surgical margins. However, the local recurrence rate in patients with OSCC is still 10–30% [4] as frozen pathology is not practical for serial sections to acquire pathology information for entire specimens during surgery. These deficiencies may lead to missed cancer cells [5]. Although more extensive resection promises lower recurrence, it may also result in increased physiological dysfunction, such as speech, swallowing, and mastication. Therefore, real-time surveillance of surgical margins is crucial to guide the removal of all cancer cells with restrained surgical resection while preserving patient function.

In recent years, fluorescent-labeled molecular probes have provided real-time, intraoperative distinction of the molecular surgical margin between cancer and adjacent normal tissue, potentially decreasing the incidence of residual cancer cells and improving function preservation [6–8]. Among these molecular probes is indocyanine green (ICG), which has been approved by the Food and Drug Administration (FDA). After intravenous injection, ICG binds to serum proteins and behaves as a macromolecule in circulation, reflecting in its passive enrichment in tumor tissue via the enhanced permeability and retention (EPR) effect [9, 10]. The EPR effect is a delayed effect that allows nanostructures to accumulate in abnormal tissues because of their leaky vasculature and dysfunctional lymphatics. ICG accumulates in tumors and fluoresces upon near-infrared light illumination, thereby permitting tumor localization and improving traditional surgical visualization and palpation [11–13]. ICG has been used for tumor imaging and resection in the breast, colon, lung, and several other types of cancer surgeries [14–17]. ICG has also been used in neurosurgery to guide brain glioma resection and plays an important role in the maximal preservation of nerve function [18]. The ICG fluorescence of the surgical margin based on passive targeting is promising for intraoperative discrimination of tumor and normal tissue.

The present study first performed preclinical in vitro and in vivo studies using ICG near-infrared fluorescence (NIF) imaging. Quantification of ICG fluorescence intensity allowed discrimination of tumor and normal tissues. Next, ICG fluorescence was measured in clinical research to monitor surgical margins on tumor bed and specimens collected from patients with OSCC using an NIF imager during surgery. Residual cancer cells were detected intraoperatively in surgical margins. All patients with OSCC underwent tumor resection 6–8 h after ICG injection.

## Materials and methods

### ICG administration and NIF imaging instrument

ICG was purchased from YiChuang Pharmaceutical Co., Ltd. (Dandong, China). NIF imaging and fluorescent intensity were determined using an NIF imaging-guided instrument (REAL-IGS, NuoYuan Medical Devices Co., Ltd, Nanjing, China) integrated with a hand-held NIF spectrometer (Maya 2000 Pro, Ocean Optics, Dunedin, FL, USA). During surgery, ICG excitation was detected at 785 nm. For NIF imaging, three sub-windows on the monitor showed fluorescence including white-light, merged, and gray images acquired by the Fluorescence Navigation Software (NuoYuan Medical Devices Co., Ltd, Nanjing, China). The hand-held NIF spectrometer was used for fluorescent intensity quantification. The measurement distance was fixed at 5 cm, and the measurement angle was perpendicular to the tissue surface. Spectra Suite (Omni Driver, Ocean Optics, Dunedin, FL, USA) was used to measure the fluorescence intensity.

### Cell imaging

The human OSCC cell lines HSC3, CAL27, and SCC4 and murine OSCC cell line SCC7 were obtained from Fudan University (Shanghai, China). All cell lines were cultured in DMEM supplemented with 10% (v/v) fetal bovine serum, 1% (v/v) penicillin, and 1% (v/v) streptomycin. Cells were incubated in a humidified incubator at 37 °C with 5% CO_2_. The cell lines were regularly tested and maintained negative for *Mycoplasma* spp. For imaging, 0.5 mL containing 5 × 10^5^ HSC3, CAL27, SCC4, or SCC7 tumor cells were seeded on the middle of six-well plates; 4 h later, after the tumor cells had adhered to the well surface, the media in each well was replaced with 3 mL fresh media with 1, 0.5, 0.25, or 0.125 mg/mL ICG. After 24 h, the treated cells were washed twice with phosphate-buffered saline (PBS) and visualize using an NIF imaging-guided instrument (REAL-IGS, NuoYuan Medical Devices Co., Ltd, Nanjing, China).

### Murine model imaging

BALB/c athymic nude mice were provided by the Comparative Medical Center of Yang Zhou University. The 48 mice were divided into eight groups, with six mice per group and injected with 1 × 10^6^ HSC3, CAL27, SCC4, or SCC7 cells implanted subcutaneously into the tongue and back, respectively. When the tumor was visible to the naked eye, the mice were intravenously injected with ICG (5 mg/kg). After 12 h, the treated mice were imaged using a NIF imaging-guided instrument. The tumor resections were conducted under the guidance of NIF imaging. All animal procedures were approved by the Animal Protection Committee of Nanjing University.

### Clinical trails

This study included 20 patients with OSCC. Patients with a history of severe hepatic and renal dysfunction were excluded. Informed consent was obtained from all patients. Patient characteristics are listed in Table 1.

**Table 1:** Clinical data

Patient characteristics (*n* = 20)	Absolute no.	Relative %
Sex		
Female	8	40.0
Male	12	60.0
Localization		
Tongue	8	40.0
Buccal mucosa	5	25.0
Palate	2	10.0
Gingival	2	10.0
Lip	1	5.0
Oral floor	2	10.0
T classification		
T2	12	60.0
T3	5	25.0
T4	3	15.0
	Mean ± SD	Median, range
Age, years	60 ± 9	60, 48–75

**Fig. 1:**
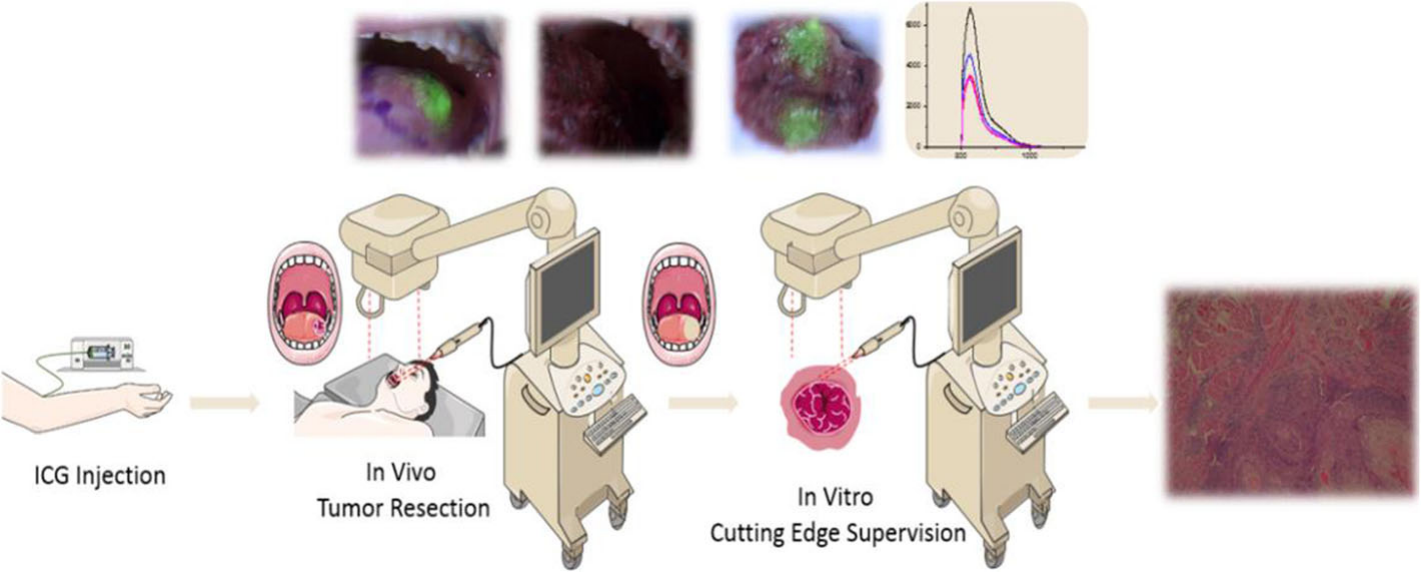
Schematic diagram of real-time intraoperative surveillance of surgical margins using an indocyanine green (ICG) near-infrared fluorescence instrument

The ICG injection dose used in this study was 0.75 mg/kg. ICG was diluted in 30 mL distilled water and injected into the antecubital vein via an intravenous pump over 30 min before imaging. All patients underwent NIF imaging surgery 6–8 h after injection. Before NIF imaging, the surgeon determined the tumor margin by visual inspection and palpation. NIF imaging was then performed, and the ICG fluorescence intensities of the tumor, peritumoral, and normal tissues were documented in vivo and in vitro. ICG fluorescence in the tumor tissues was measured at the 3, 6, 9, and 12 o’clock positions. The average fluorescence intensity was defined as tumor tissue signals. ICG fluorescence detected in tissues on the surgical margin was defined as peritumoral tissue signal. ICG fluorescence detected in tissues away from the tumor in the oral cavity was defined as normal tissue signal. After tumor resection, the fluorescent signals of the surgical margins of the wound and specimens were measured by NIF imaging device to identify residual cancer cells. When ICG fluorescence or abnormal fluorescence intensity were detected, tissues from these areas were re-resected and pathological examination was performed to confirm the presence of residual cancer cells (Fig. 1)

### Statistical analysis

Statistical analysis was performed using IBM SPSS Statistics for Windows, version 23.0 (IBM Corp., Armonk, NY, USA). Values are presented as means ± standard deviation. Signal-to-background ratio (SBR) was applied to quantify fluorescence contrast. One-way analysis of variance was used to compare the fluorescence intensities of tumor, peritumoral, and normal tissues. *P* < 0.05 was considered statistically significant.

## Results

### ICG localizes to tumor cells in vitro and tumors in mice

**Fig. 2:**
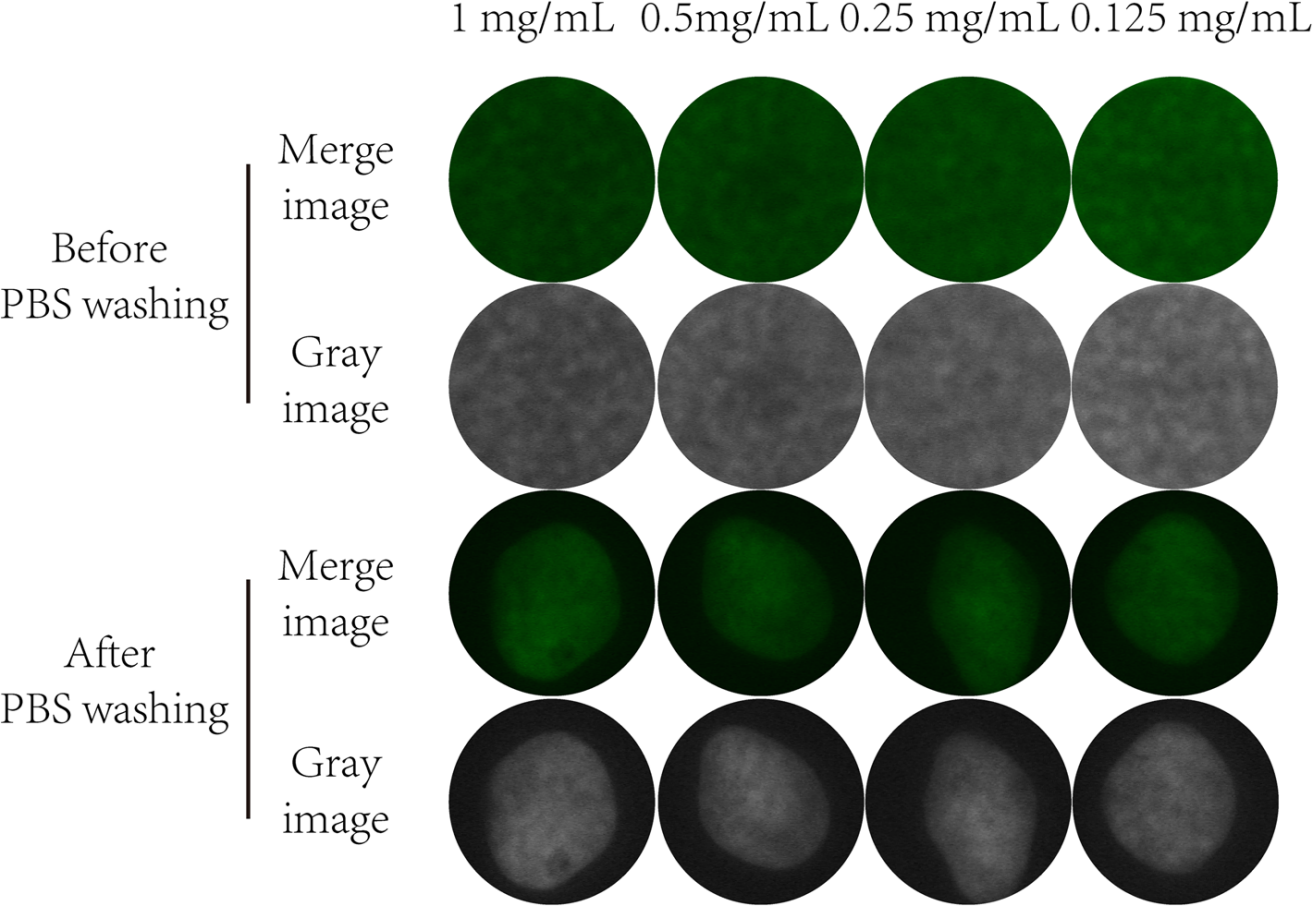
Cell imaging with different ICG concentrations

**Fig. 3:**
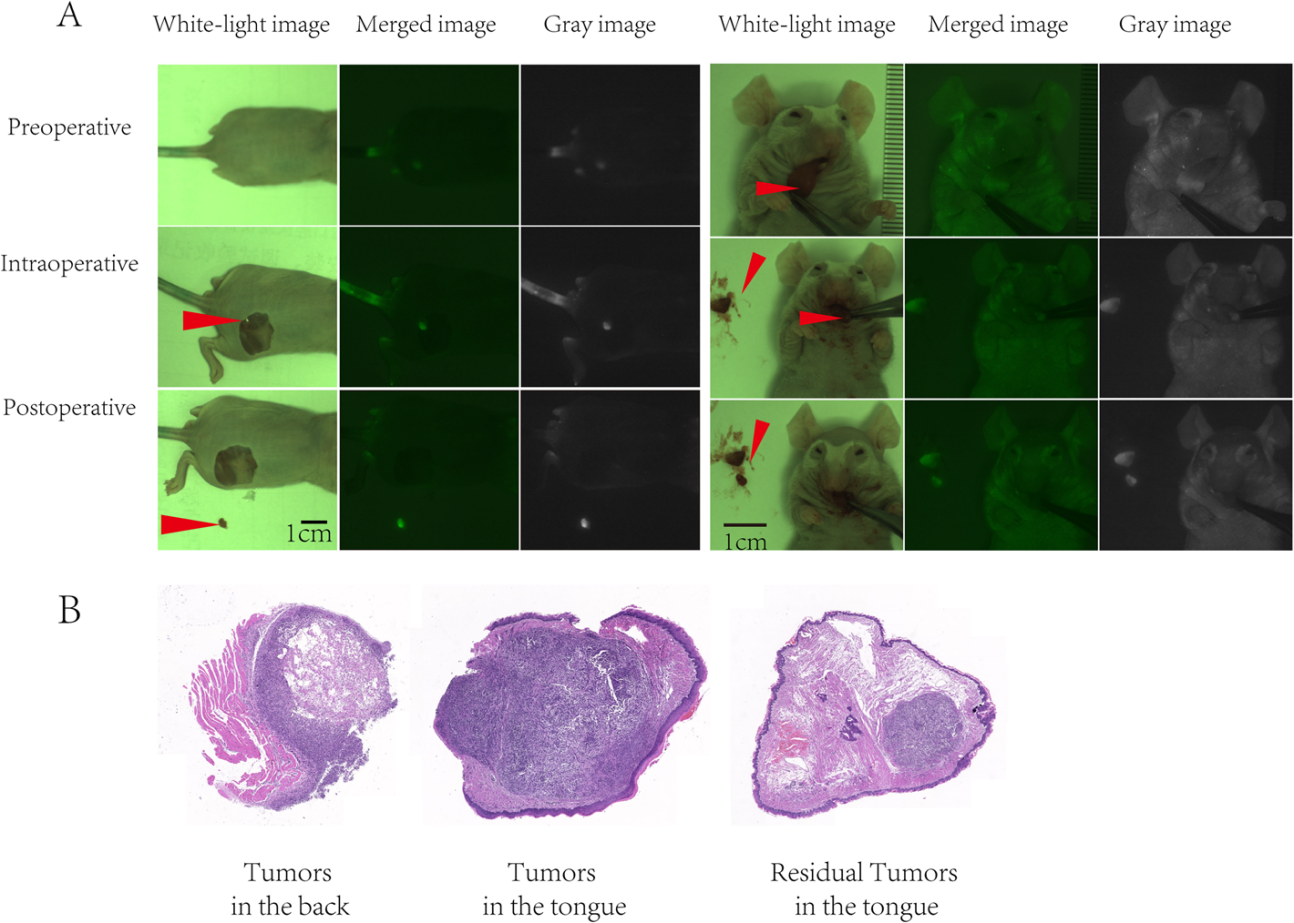
Pre-clinical studies of surgical margin monitoring by near-infrared fluorescence (NIF) imaging and spectroscopy in oral squamous cell carcinoma (OSCC). **a** NIF imaging surgery stimulation in OSCC subcutaneously implanted tumor and orthotopic transplantation models. **b** Hematoxylin and eosin-stained tumor sections

In vitro and in vivo experiments were conducted to evaluate the feasibility of monitoring surgical margins via NIF imaging and spectroscopy in OSCC. For in vitro imaging, we incubated cells with different concentrations of ICG solutions. Twenty-four hours later, all the regions of tumors showed fluorescence at ICG doses of 0.125–1 mg/mL (Fig. 2). This result reminded us that OSCC tumor cells could uptake ICG and produce fluorescence in vitro. For in vivo imaging, both subcutaneously implanted tumor and orthotopic transplantation models were constructed. Twelve hours after ICG injection, tumor resection under NIF imaging showed fluorescence in tumor regions in all 48 mice. The average tumor volume was 9.51 ± 9.74 mm^3^, with a minimum volume of less than 0.5 mm^3^. The average fluorescence intensities of the tumor and normal tissues were 123.2 ± 5.6 and 41.2 ± 7.8 AUs, respectively. The SBR of tumor to normal tissues were computed to be 3.1 ± 0.67. In addition, after complete tumor removal, no fluorescence was detected in the tumor bed; however, abnormal fluorescence was detected in mice with incomplete tumor removal (Fig. 3a, b).

### ICG localizes to tumor tissues in patients with OSCC

**Fig. 4:**
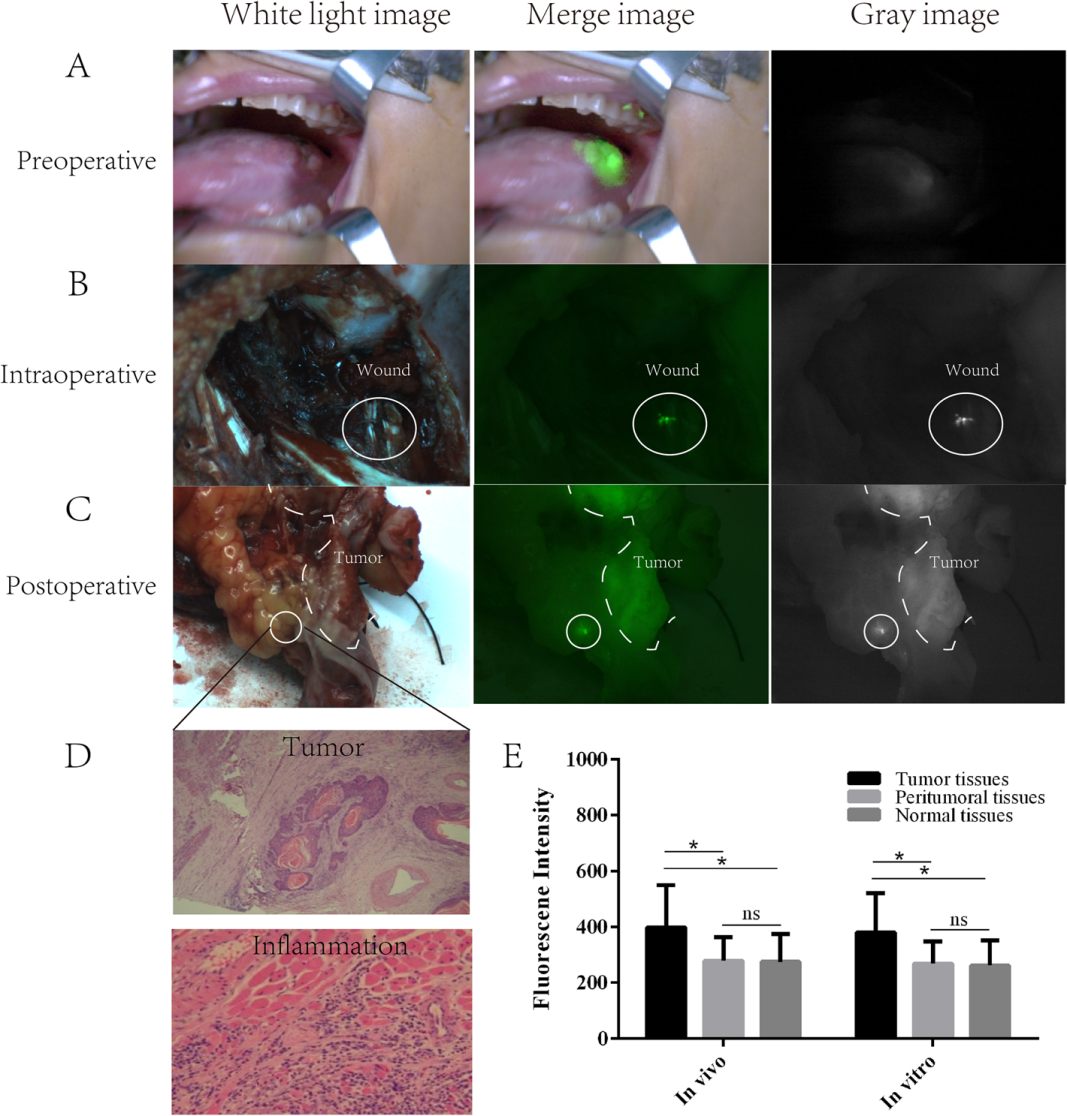
Clinical trials of monitoring of surgical margins by near-infrared fluorescence (NIF) imaging and spectroscopy in oral squamous cell carcinoma (OSCC). **a** Preoperative indocyanine green (ICG)-based NIF imaging of OSCC tumor tissues. **b** Intraoperative ICG-based NIF imaging of OSCC tumor beds. **c** Postoperative ICG-based NIF imaging of OSCC tumor specimens. **d** Pathological results of tissues with fluorescence developed. **e** Fluorescence intensities of tumor, peritumoral, and normal tissues in vivo and in vitro

After confirming the feasibility of discriminating tumor tissues from normal tissues in the murine OSCC model, we conducted the clinical trials. At 6–8 h after the intravenous administration of ICG, all tumors showed ICG fluorescence, clearly delineating the tumor border from normal tissues via NIF instrument in all 20 patients (Fig. 4a). The average fluorescence intensities of tumor, peritumoral, and normal tissues on the hand-held NIF spectroscopy device were 398.863 ± 151.47, 278.52 ± 84.89, and 274.5 ± 100.93 AUs, respectively (Fig. 4e). The SBR of tumor to peritumoral tissue and normal tissues was computed to be 1.45 ± 0.36 and 1.56 ± 0.41, respectively. The fluorescence intensity of tumor tissues was significantly high than those of peritumoral and normal tissues (*P* < 0.05). After tumor resection under NIF imaging, the NIF image instrument was used to re-examine the tumor beds for residual cancer cells. Abnormal ICG fluorescence was detected in four patients (4/20) (Fig. 4b). Additional excision was performed at these sites. Residual cancer cells were confirmed by the pathologist, while inflammatory cell and not cancer cell infiltration was confirmed at the sites of fluorescence cells in the remaining two patients (Fig. 4d). Next, the in vitro specimens were halved and observed with the NIF instrument (Fig. 4c). The average fluorescence intensities of the tumor, peritumoral, and normal tissues were 380.15 ± 141.24, 268.52 ± 79.12, and 262.12 ± 90.16 AU, respectively (Fig. 4e). The SBR of tumor to peritumoral tissue and normal tissues was computed to be 1.38 ± 0.22 and 1.43 ± 0.27, respectively. Abnormal fluorescence was also detected in the same four patients, two of which were confirmed to have positive surgical margins by the pathologist (Fig. 4d).

## Discussion

In head and neck oncology, ICG NIF imaging has been introduced for sentinel lymph node mapping and evaluation of mucosal head and neck lesions [19, 20]. For ICG-based tumor imaging, Yokoyama et al. [21] were the first to observe five patients with oral or oropharyngeal cancers. The authors reported an ICG sensitivity of 100%. Other reports showed ICG positivity in 36 patients with advanced paranasal sinus cancer [22]. These results confirmed that ICG was an excellent molecular tracer for head and neck cancers, in addition to other solid tumors. In our study, all 20 patients with OSCCs were ICG-positive. ICG fluorescence tumor borders were distinct on grayscale imaging. Tumor fluorescence was detected intraoperatively using the ICG NIF imager. Pathological examination confirmed that 18 patients (18/20) had no remaining cancer cells after tumor resection. The results of our study proved that ICG injection accompanied by NIF imaging provided a feasible and effective way to inspect surgical margins during OSCC surgery.

Precise tumor margin identification and radical tumor resection during surgery are crucial for cure in many types of tumors [23, 24], including oral cancer. Imaging and clinical examinations are commonly used in the determination of tumor margins at present, but they are not always sufficient for the discrimination between tissue types and can lead to irrational resections. In breast cancer, for example, many of which are non-palpable, the margin positivity rates range from 5 to 49% [25, 26]. Due to the complex oral anatomy, such as inter-connected tissue space, it is more difficult to determine tumor margins by the naked eye or palpation in surgery. Intraoperative ICG NIF imaging has been used for lung, breast, liver, and other cancers [16, 27–31]. Satou et al. [30] reported the detection of five extrahepatic metastases that were otherwise not detected in two of 17 patients (11.7%) with the help of NIR fluorescence imaging. Yokoyama et al. [31] demonstrated that NIF imaging after ICG injection identified hepatic metastases of pancreatic carcinoma that were otherwise not detected in eight of 49 patients (16.3%). In our study, ICG NIF imaging helped to detect positive surgical margins in two of 20 patients (10%), which would have otherwise been missed. Thus, 10% of the patients with oral cancer in this study might be free from local tumor recurrence. The above studies confirmed that NIF fluorescence imaging has complementary value during radical tumor resection.

Evaluation of surgical margins for OSCC includes both mucosal and deep soft tissue margins. However, safe surgical margins are hard to determine in deep soft tissue during surgery. In our study, the NIF imager and handheld spectroscopic probe showed ICG-positive fluorescence or abnormal fluorescence intensity in wounds and specimens in four patients. Additional resection was performed in these patients. Residual cancer cells were identified in the deep soft tissue margins (the medial pterygoid and mylohyoideus muscles) of two of these patients (with gingival and oral floor cancer, respectively), which were further confirmed by the pathologist. The remaining two patients had false-positive results. These findings may be related to the fact that inflammation can similarly accumulate ICG via the EPR effect. Our results showed the utility of ICG NIF imaging to assess the surgical margins of deep soft tissue in patients with OSCC.

This study used spectrometry and grayscale imaging for real-time surveillance of surgical margins. Spectrometry was superior at detecting minimal fluorescence changes between cancer cells and normal tissues. The sensitivity of the NIF instrument was 0.01 μM for ICG in vitro according to our previous studies. Thus, spectrometers provide an ideal intraoperative approach to detect residual cancer cells in surgical margins [32]. However, despite its lower sensitivity, grayscale imaging was significantly easier to use intraoperatively than spectrometry. The ability of a surgeon to visualize fluorescence in a wide field of view allows rapid interrogation and evaluation of the entire organ surface [33]. Molecular imaging can detect up to 50% more residual tumor deposits compared to traditional margin detection methods, providing a 50% higher recurrence-free survival rate [34]. Our findings demonstrated the intraoperative identification of residual cancer cells by ICG NIF imaging in 2/20 patients. The combined use of spectrometer and grayscale imaging offers more effective intraoperative detection of tumor cells.

## Conclusion

While visual inspection and finger palpation remain the fundamental skills of a surgeon, we show that, ICG NIF imaging serves as a promising supplement in surgery to guide tumor resection and monitor surgical margins, thus to improve patient clinical outcomes and reduce overall health-care cost.

## Supplementary information

**Additional file 1: Fig. S1.** Verification of the consistency between fluorescence boundary and tumor boundary in intraoperative frozen section.

## Data Availability

The dataset supporting the conclusions of this article are included within the article and its additional file.

## Abbreviations

AUs: Arbitrary units
EPR: Enhanced permeability and retention
FDA: Food and Drug Administration
ICG: Indocyanine green
NIF: Near-infrared fluorescence
OSCC: Oral squamous cell carcinoma
PBS: Phosphate-buffered saline
SBR: Signal-to-background ratio
