# Supplementary material for: Real-time surveillance of surgical margins via ICG-based near-infrared fluorescence imaging in patients with OSCC
Source: World J Surg Oncol. 2020 May 15;18:96. doi: 10.1186/s12957-020-01874-z (PMC7229610; doi:10.1186/s12957-020-01874-z)
Supplement: Supplementary file 1 — Additional file 1: Fig. S1. Verification of the consistency between fluorescence boundary and tumor boundary in intraoperative frozen section. [file 12957_2020_1874_MOESM1_ESM.docx]

Support information


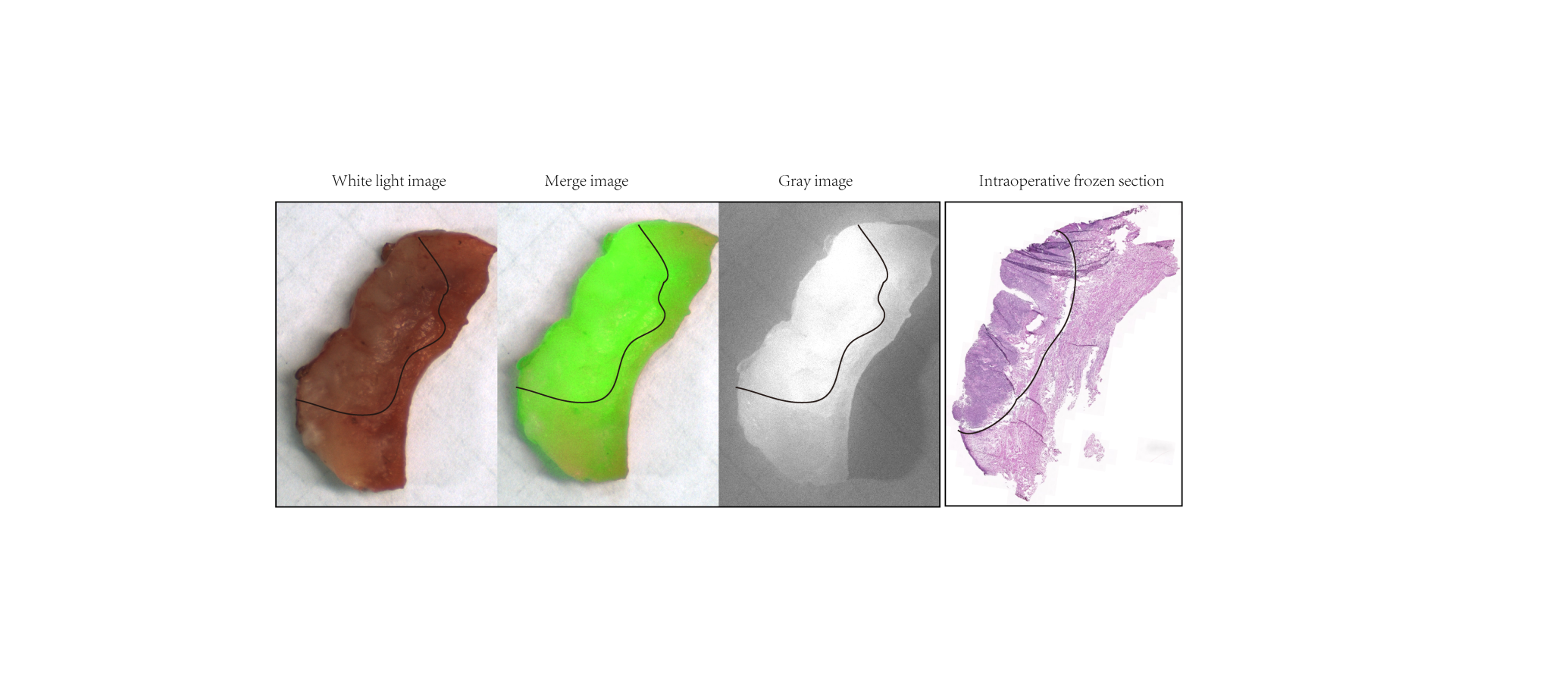


Fig.S1 Verification of the consistency between fluorescence boundary and tumor boundary in intraoperative frozen section.

Firstly, the *in vitro* tumor tissue samples were imaged with NIF instruments to delineating the tumor border from normal tissues. Then a sharp scalpel was used to separate the tumor tissues and normal tissues along the fluorescence boundary as shown in the figure. At last, the separated tissues were performed with intraoperative frozen section and H&E staining.

From the intraoperative frozen section, we could found the tumor boundary is consistent with the fluorescence boundary.
